# 3D vessel-wall virtual histology of whole-body perfused mice using a novel heavy element stain

**DOI:** 10.1038/s41598-018-36905-z

**Published:** 2019-01-24

**Authors:** P. Joy Dunmore-Buyze, Charmainne Cruje, Zengxuan Nong, Jason J. Lee, John A. Kiernan, J. Geoffrey Pickering, Maria Drangova

**Affiliations:** 1 Robarts Research Institute, The University of Western Ontario, London, Ontario N6A 5B7 Canada; 2 Department of Medical Biophysics, The University of Western Ontario, London, Ontario N6A 5C1 Canada; 3 Department of Anatomy and Cell Biology, The University of Western Ontario, London, Ontario N6A 5C1 Canada; 4 Department of Physiology and Pharmacology, The University of Western Ontario, London, Ontario N6A 5C1 Canada; 5 Departments of Medicine and Biochemistry, The University of Western Ontario, London, Ontario N6A 5B7 Canada

## Abstract

Virtual histology – utilizing high-resolution three-dimensional imaging – is becoming readily available. Micro-computed tomography (micro-CT) is widely available and is often coupled with x-ray attenuating histological stains that mark specific tissue components for 3D virtual histology. In this study we describe a new tri-element x-ray attenuating stain and perfusion protocol that provides micro-CT contrast of the entire vasculature of an intact mouse. The stain – derived from an established histology stain (Verhoeff’s) – is modified to enable perfusion through the vasculature; the attenuating elements of the stain are iodine, aluminum, and iron. After a 30-minute perfusion through the vasculature (10-minute flushing with detergent-containing saline followed by 15-minute perfusion with the stain and a final 5-minute saline flush), animals are scanned using micro-CT. We demonstrate that the new staining protocol enables sharp delineation of the vessel walls in three dimensions over the whole body; corresponding histological analysis verified that the CT stain is localized primarily in the endothelial cells and media of large arteries and the endothelium of smaller vessels, such as the coronaries. The rapid perfusion and scanning protocol ensured that all tissues are available for further analysis via higher resolution CT of smaller sections or traditional histological sectioning.

## Introduction

Advances in vascular research rely heavily on detailed characterization of rodent models of disease. Histology is commonly employed to investigate the architecture and constituents of the vasculature and organs of mice^1–3^ and study various disease models^4–7^. Histological protocols require the excision of tissue followed by fixation, dehydration, clearing and embedding, cutting into thin sections (5–10 μm) and immersing in chemical stains for varying periods of time. Although standard histology provides images with high resolution, it is limited by the requirement for processing a limited number of sections, thereby losing three-dimensional context. Furthermore, traditional histological staining protocols are time-consuming and are prone to introducing morphological artifacts^8^. A technique that enables the microanatomy of rodents to be studied in three dimensions (3D) while leaving organs of interest intact would allow for structural and morphological information to be obtained^9–13^.

Micro-computed tomography (micro-CT) is a cost-effective and widely available 3D imaging technique that provides an opportunity to non-destructively study intact rodents and explanted tissues at high resolution. Recently, a strategy of using histological stains that mark specific tissue components combined with micro-CT has been applied in “virtual histology” – a technique that can be used to study the microstructure of tissues without disturbing the morphology of an organ or tissue of interest. Several groups have used high resolution micro-CT for virtual histology to visualize soaked excised organs and tissues in iodine-potassium iodide (I_2_KI)^14–20^, phosphotungstic acid (PTA)^8,9,21^, and osmium tetroxide (OsO_4_)^22^ for periods of time ranging from hours to weeks. Previous reports have also shown that unstained tissue can be visualized using other x-ray based imaging techniques, such as phase contrast CT^8^ and photon counting^23^. However, these techniques have limited field of view and require the removal of tissue from its native anatomy. Since the vascular system is a network that encompasses the whole body, there is great value in observing the gross vasculature in addition to imaging select tissues. Our group has developed a whole-body perfusion technique, which obviates the need to extract and soak individual organs or tissues. This technique exploits the vasculature as a channel to deliver stains throughout the entire body^24^.

The previously studied stains are of interest in micro-CT imaging because they contain heavy elements *(i.e*. iodine, tungsten and osmium). Since these stains have known affinities for specific tissue components – such as PTA for collagen^25–27^, fibrin^3^, muscle fascicles^16^, and extracellular tissue proteins in the glomeruli^9^ or I_2_KI for soft tissues such as myocardial muscle fibres^28^ and various connective tissues^17,18,29^ – their presence in micro-CT images would coincide with the abundance of the tissue component it selectively stains. For models of vascular disease, stains that bind to endothelial cells, collagen, extracellular matrix proteins and elastin would be advantageous. While I_2_KI, PTA, and OsO_4_ are useful in studying the vessel wall as a whole unit, no micro-CT stain that highlights individual wall components has been demonstrated. A commonly used histology stain for elastic tissue and cell nuclei is Verhoeff’s stain, which is reported to bind to elastin by van der Waals forces^30–32^. Verhoeff’s stain contains two x-ray attenuating materials (iron and iodine), making it an attractive candidate as a micro-CT stain for vascular analysis.

In this study, we describe a novel, tri-element x-ray attenuating stain and perfusion protocol derived from Verhoeff’s stain that provides micro-CT contrast of the entire vasculature of an intact mouse. Rapid perfusion – of less than 30 minutes – with the novel stain results in micro-CT images of vessel walls, which compared favourably with images of I_2_KI- and PTA-perfused mice. Corresponding histological sections localized the heavy elements within the vessel wall. We describe our novel stain and demonstrate its ability to provide 3D images of vessel walls throughout the entire mouse vasculature, and hence its utility in virtual histology; to our knowledge, no other CT staining technique localizes contrast within the structural components of the vessel wall, thereby producing whole-mouse vasculature-specific images using 3D micro-CT, following a perfusion procedure under 30 minutes.

## Materials and Methods

### Animal handling and preparation

Animal studies were performed under a protocol approved by the University Council on Animal Care at the University of Western Ontario, which follows the policies set out by the Canadian Council on Animal Care. The “CT stain” perfusion techniques presented in the paper were developed using 10 mice (C56BL/6 male, 25–30 g, Jackson Laboratory, Bar Harbor ME) for each stain studied; results are reported from two mice for each of the CT stains. Prior to cannulation of the abdominal aorta, mice were anaesthetized with an intraperitoneal injection of 50 mg/kg of ketamine hydrochloride and 12.5 mg/kg of xylazine; to prevent clotting during the perfusion, 0.2 mL of heparin (Sodium Injection USP, 200 units, Sandoz Canada Inc.) was injected intraperitoneally. A midline incision was made in the abdomen, beginning just below the diaphragm. The abdominal aorta was dissected free and cannulated using a 24 GA, 19 mm long I.V. catheter; the catheter was attached to the perfusion system (see below) and the inferior vena cava was cut to allow drainage of the perfusate.

### CT stains

Three different CT stains were evaluated. All solutions were filtered prior to perfusion and were used within 24 h of preparation in de-ionized water. Following prior work^24^ 5% w/v PTA and I_2_KI (2.5% w/v KI and 1.3% w/v I_2_) solutions were prepared. A modified Verhoeff’s solution was also prepared, where the modification consisted of replacing the traditional alcoholic heamatoxylin in the formulation with an aluminum-heamatoxylin solution (Surgipath, Harris Hematoxylin, Leica Biosystems). The stain components were modified because the traditional Verhoeff’s stain^33^ (containing 5% w/v heamatoxylin in ethanol) caused precipitation when perfused through the animal and prevented complete perfusion by occluding small vessels. Specifically, the modified solution contained 56% v/v aluminum (Harris) heamatoxylin, 2.2% w/v FeCl_3_•6H_2_O, 0.6% w/v KI and 0.3% w/v I_2_.

### Perfusion system

Consistency of the perfusions was facilitated by the use of a modified ValveBank®8 Pinch Valve (Automate Scientific, Berkeley, CA) gravity-fed perfusion system, which seamlessly switches between solutions (Supplementary Fig. S1). The pinch valve apparatus of the commercial system was redesigned to allow for the use of larger diameter tubing (4.76 mm), which provided the flow rates and pressure required to perfuse whole mice and rats. Up to 6 different solutions can to be perfused, as required, without moving the animal once the catheter is in place.

### Saline flush with and without a detergent

Clearing of the blood prior to perfusion with a stain was achieved by perfusing the vasculature with heparinized saline (0.9% NaCl; 0.1% heparin). To increase the permeability of the vessel wall, a second solution was prepared by adding a non-ionic detergent (0.03% Triton™ X-100, BDH Analytical Chemicals) in phosphate buffered saline.

### CT-stain perfusion protocol

Mice were perfused via retrograde perfusion of the abdominal aorta at physiological pressure (~110 mm Hg). Heparinized saline – with or without Triton™ X-100 – was flushed through the vasculature for 10 minutes, which caused animal death by exsanguination. One of the three CT stains was then delivered using the following timing: 15 minutes for I_2_KI, 30 minutes for PTA, and 10 minutes for the aluminum-modified stain. These times were based on earlier work and preliminary tests aimed at optimizing vessel contrast (aluminum-modified CT stain). Finally, the vasculature was flushed with normal saline for 5 minutes. We will refer to the aluminum-modified stain applied following saline flush as AlumHemFeI and to the stain applied following flushing with saline with Triton™ X-100 as AlumHemFeI-T. Additional mice were perfused with heparinized saline – with or without Triton™ X-100 – for 15 minutes to act as negative controls.

### Micro-CT imaging

Immediately following perfusion, whole body micro-CT scans were obtained on a GE eXplore speCZT scanner (GE Healthcare, London, ON) using a 5-minute protocol (900 views, 0.4° angle increment). Scan parameters were 90 kVp, 40 mA, 2 × 2 binning, and 16 ms exposure time. Images were reconstructed with 50 µm isotropic voxels and intensities were converted to Hounsfield units (HU), where the image intensities scale linearly with attenuation and the value of water equals 0 HU and that of air equals −1000 HU. Following micro-CT scanning, tissue samples were harvested for histology.

**Figure 1:**
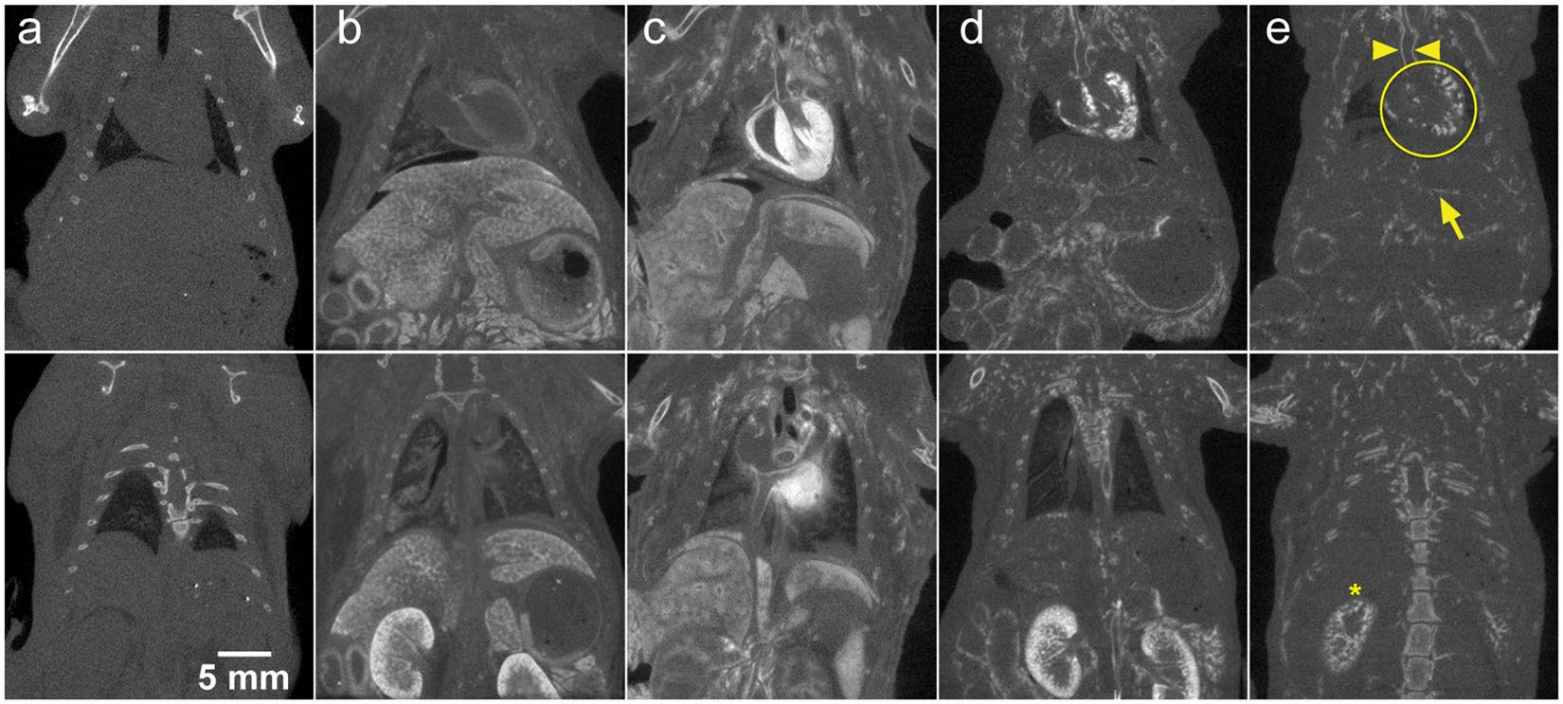
Coronal slices from micro-CT volume images (50 µm isotropic voxels) of whole-body perfused mice. The images illustrate the difference in contrast enhancement based on the stain perfused: (**a**) saline, (**b**) PTA, (**c**) I_2_KI, (**d**) AlumHemFeI and (**e**) AlumHemFeI-T. The image slices shown were selected to demonstrate uptake by the aorta (arrowheads), heart (circle), and the liver (arrow) in the top row, and kidneys (asterisk) in the bottom row, which are labeled in (**e**) for reference.

### Image visualization and analysis

The micro-CT images were viewed and analysed using MicroView (Parallax Innovations, London, ON). While the general “binding affinity” of the CT stains is consistently reproducible, absolute increases in x-ray attenuation is variable between specimens and tissue types. Nevertheless, quantitative evaluation of the attenuation (in HU) of representative tissues is valuable^27^ and was performed for two mice perfused with each CT stain. Because the murine vessel wall is less than 100 µm thick, regions of interest were defined as 0.5-mm (10-voxel) long single-voxel line profiles drawn along the length of the ascending aorta in three consecutive slices (all stains) and along select coronary arteries (AlumHemFeI and AlumHemFeI-T only); for each region the mean and standard deviation were calculated. (In essence, the regions of interest represent 3 × 10 × 1 voxel patches of the vessel wall.) For reference, similar regions of interest were defined in the humerus (forelimb), which is representative of cortical bone. Because the entire vasculature is perfused during the experiment, unstained soft tissue cannot be identified consistently in the mice and therefore no attempt was made to calculate contrast enhancement over background. Lastly, for comparison to prior work^24^, 0.5 × 0.5 × 0.5 mm regions of interest were used to measure attenuation in the myocardium of the PTA- and I_2_KI-stained mice. The measured values were averaged to generate a mean CT number for each tissue type. The mean of the standard deviations of the regions of interest from the two mice are also reported; these standard deviations represent a combination of image noise and variability in regional stain delivery.

### Histological analysis

In order to identify the vessel-wall components to which the various chemical agents may be adhering to, histological processing tailored to each stain compound was performed. The I_2_KI-perfused tissues were removed from the perfused animal and embedded in OCT so that frozen sections could be obtained; these frozen sections were examined with no additional processing or staining because I_2_KI appears brown in the sections and is largely removed by water or organic solvents. Samples from the PTA, AlumHemFeI and AlumHemFeI-T-perfused animals were fixed in 4% paraformaldehyde overnight, embedded in paraffin, and sectioned (5 µm thick). Toluidine blue^34^, which combines with PTA to form an insoluble pigment, was used to counter-stain the colourless PTA-perfused samples. The iron-containing AlumHemFeI- and AlumHemFeI-T-stained sections were counter-stained with a Perls’ Prussian blue method^35^, to localize iron in the tissues. Sections from saline-perfused mice were also stained, as negative controls for the CT stains. Sections were viewed and photographed using an Olympus BX51 (Center Valley, PA) microscope.

## Results

### Qualitative comparison of CT stains

Whole-body retrograde perfusion of CT stains was successfully – and reproducibly – achieved using the apparatus and protocols developed. The images of Fig. 1 confirm prior results, using hand perfusion, with PTA and I_2_KI. PTA weakly bound to the myocardium and enhanced the parenchymal structure of the liver (Fig. 1b). In contrast, I_2_KI clearly enhanced the myocardium and enabled visualization of myocardial structure, while the coronary artery lumina appear as voids (Fig. 1c). I_2_KI also bound to the walls of the aorta and liver vessels, but it diffused into the tissue surrounding the vessels. The AlumHemFeI stain, when perfused following saline flushing, demonstrated a great affinity for arterial wall, particularly that of the coronaries, renal arteries, aorta, and carotids. However, the stain also appeared to diffuse through the wall and enhance surrounding tissues, such as the myocardium and renal cortex (Fig. 1d). The inclusion of Triton™ X-100 in the saline flush, results in a clearer visualization of the arterial wall of all major organs with minimal diffusion into surrounding tissue (Fig. 1e). When the four staining protocols are compared, it is evident that the AlumHemFeI and AlumHemFeI-T provide delineation of the aortic wall better than that seen with PTA, comparable to that seen with I_2_KI, and providing, in addition, images of coronary, renal, and hepatic vessel walls.

**Figure 2:**
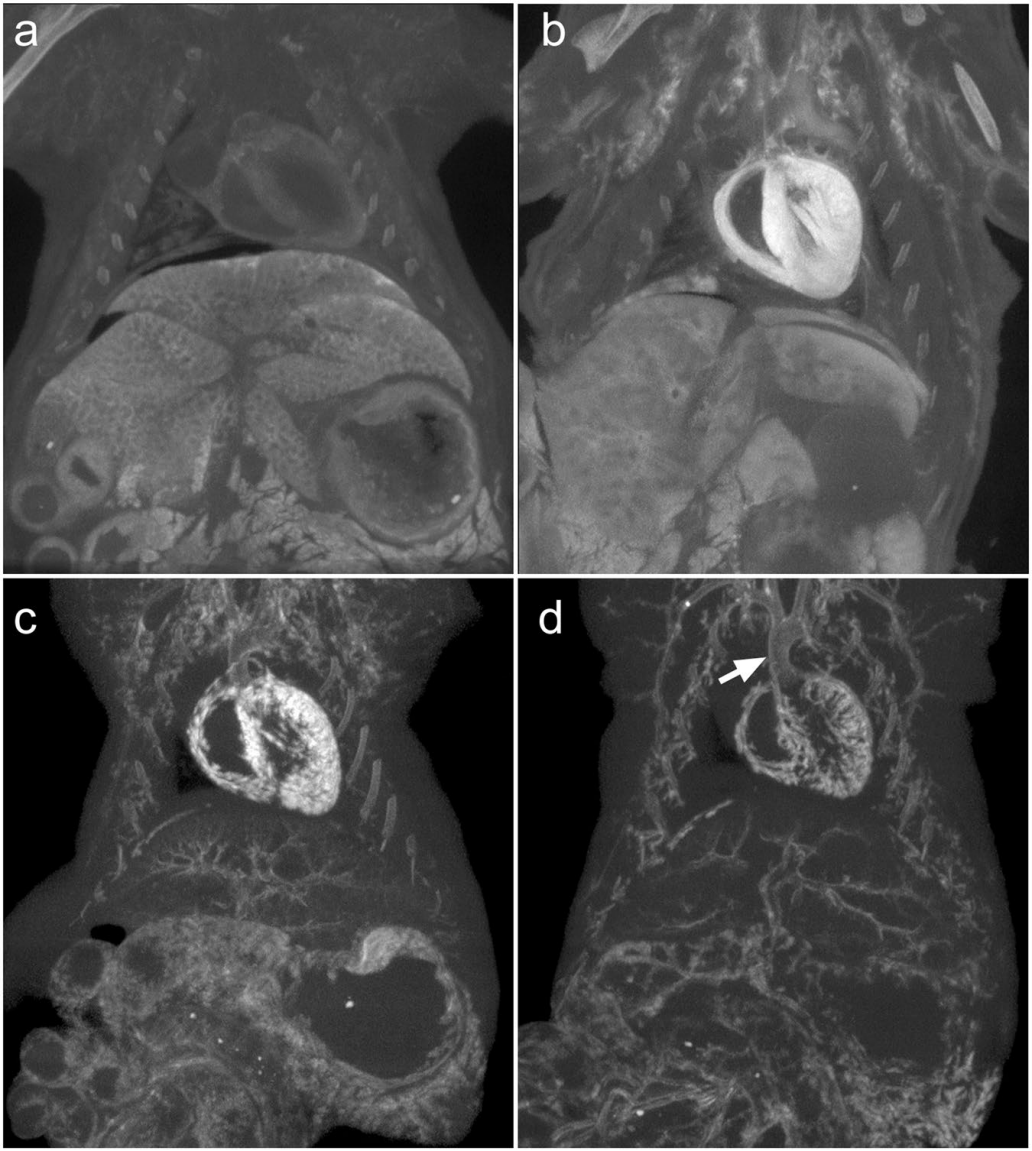
Thick maximum intensity projections (1 mm thick) of the thorax and abdomen of mice perfused with the 4 different stains: PTA (**a**), I_2_KI (**b**), AlumHemFeI (**c**), and AlumHemFeI-T (**d**). It can be seen that the AlumHemFeI-T stain provides a clear delineation of arterial walls; the arrow points to the aortic arch.

The effectiveness of AlumHemFeI-T in delineating the vessel wall is further demonstrated by observing the thick maximum intensity projections (MIPs, 1-mm thick) in Fig. 2. Whereas PTA and I_2_KI stains marked whole organs, AlumHemFeI-T displayed a clear delineation of the vasculature feeding and within these organs.

**Figure 3:**
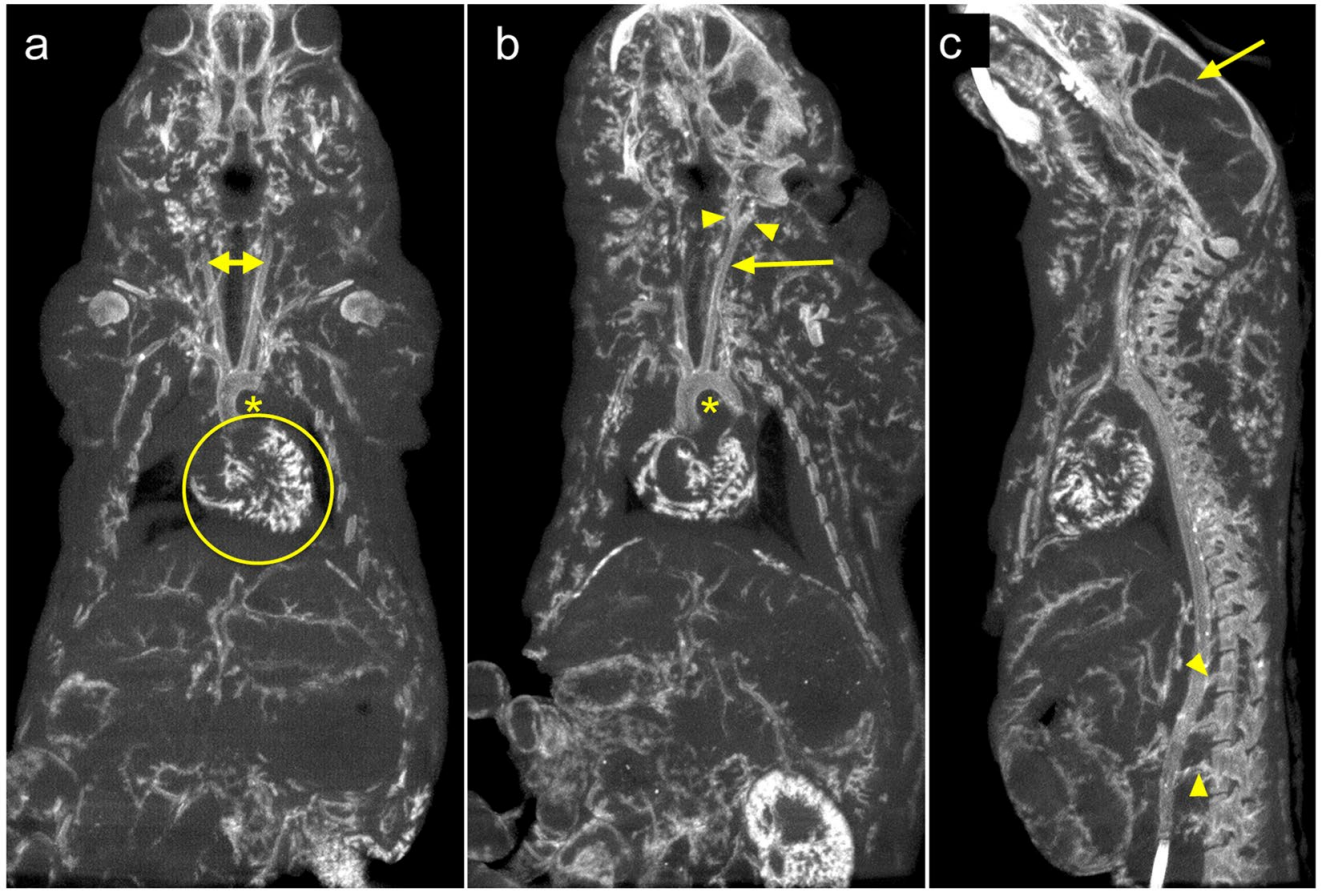
Coronal (**a**,**b**) and sagittal (**c**) slices of an AlumHemFeI-T whole-body perfused mouse. Slices are taken from images acquired with a 5-minute scan protocol (90 kVp; 50 μm voxels). The images are 1-mm maximum intensity projections selected to highlight the coronary vasculature (circle in **a**), aortic arch (*in **a**,**b**), common carotid arteries (arrows in **a**,**b**), the internal and external carotid arteries (arrowheads in **b**), the cerebral vasculature (arrow in **c**), and the small lumbar vessels (arrowheads in **c**). All images can bee seen by scrolling through the Supplementary Videos SV1, SV2 and SV3.

Figure 3 shows three different thick-slab MIPs from a single mouse and provides further convincing demonstration that delivery of the AlumHemFeI-T solution resulted in concentrated and extensive staining of the vessel walls, without diffusing into the surrounding tissues. Figure 3a shows the aortic arch, coronary arteries including intramyocardial branches, and the liver vasculature. The AlumHemFeI-T stain can also be clearly seen in the common carotid arteries, the internal and external carotids, the kidney vasculature (Fig. 3b), and the small lumbar vessels in the spine as well as the cerebral vessels (Fig. 3c). The oblique MIPs of Fig. 3 and the Supplementary Videos SV1–SV3 further demonstrate the ability to follow the arterial vasculature throughout the body.

### Quantitative analysis

The results of the quantitative analysis of CT enhancement provided by the four different staining protocols are presented in Table 1. These results show that each stain binds differently depending on the tissue type. PTA generally provided weaker enhancement than the iodine-containing stains. While I_2_KI delivery yielded the highest CT numbers in the aortic wall, it did not delineate the coronary artery wall. Instead, coronary artery wall enhancement was quantifiable only in mice perfused with the AlumHemFeI and AlumHemFeI-T agents.

**Table 1:** Quantitative contrast of various tissue types; all values are in HU.

Tissue type	CT stain									
	PTA		I_2_KI		AlumHemFeI		AlumHemFeI-T		Saline	
	mean*	SD^£^	mean	SD	mean	SD	mean	SD	mean	SD
aorta	424	50	1623	72	913	30	731	23	—	
coronary					3063^§^	176	1377^§^	116	—	
myocardium	583	153	3338	124					101	37
cortical bone^†^	1968	71	1846	86	1690	151	1390	125	1452	93

^*^Mean of the regions of interest from two mice for each tissue type.

^£^Mean of the standard deviations of the regions of interest from the two mice, representing noise and variability in regional stain delivery.

^§^Higher attenuation observed in the coronary vessel walls of AlumHemFeI perfused mice compared to the AlumHemFeI-T mice is attributed to the increased localization of the stain within the vessel wall when AlumHemFeI-T is used.

^†^Cortical bone values agree with prior data^39^; variations can be attributed to differences in age.

^_^In the saline-perfused mice all non-fatty soft tissue has the same mean attenuation; no attempt was made to identify aorta or coronaries.

### Histology of CT-stained tissues

**Figure 4:**
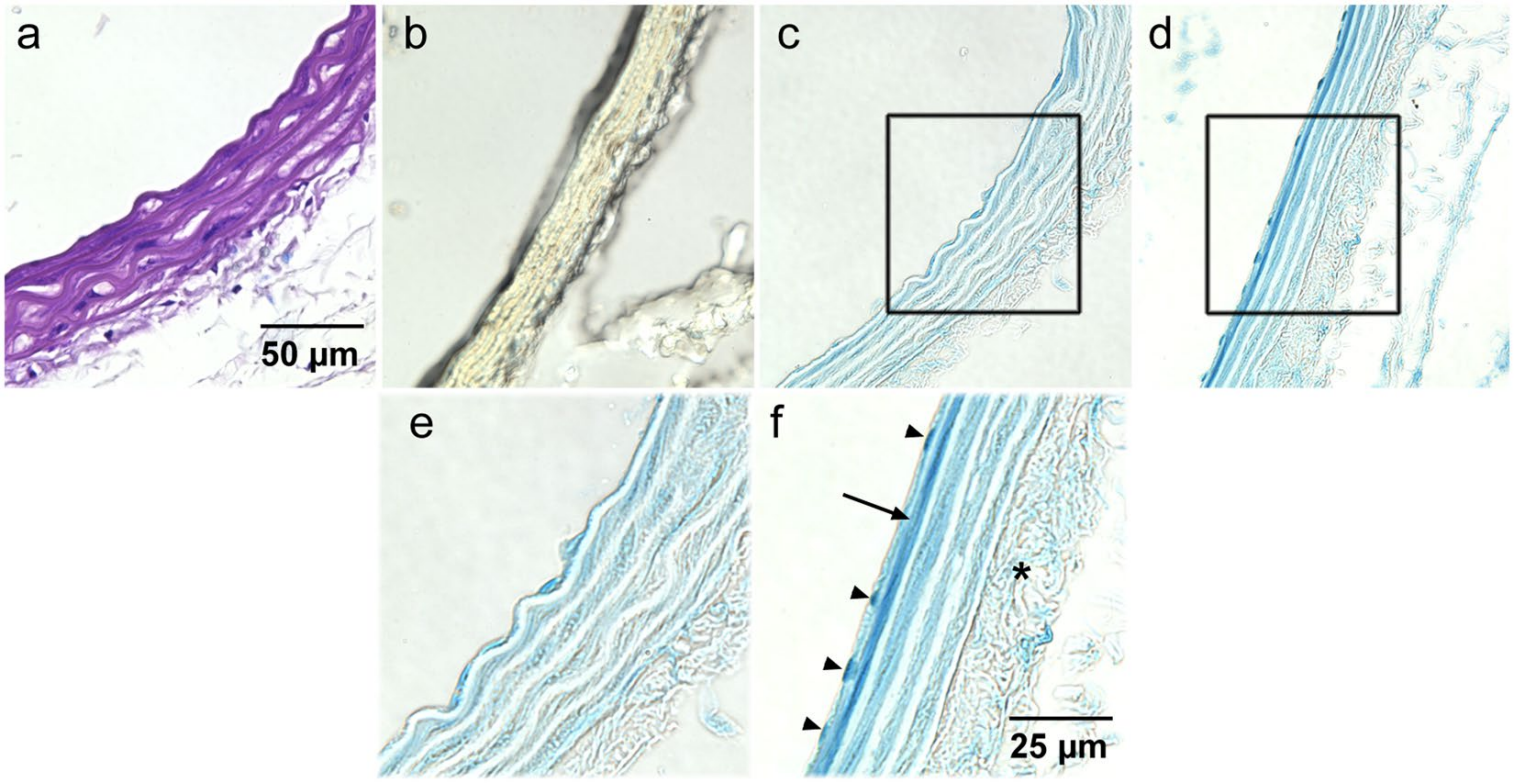
Sections of aortas from mice perfused with PTA (**a**), I_2_KI (**b**), AlumHemFeI (**c**) and AlumHemFeI-T (**d**). The images in (**e**) and (**f**) are magnifications of the regions outlined in c, and d respectively. The purple colour in (**a**) indicates the presence of PTA within the aortic wall. The iodine within the aortic wall is orange-brown in colour (frozen section). Both AlumHemFeI (**c**,**e**) and AlumHemFeI –T (**d**,**f**) stain the interlamellar spaces, but AlumHemFeI-T provides more intense staining of the endothelium (arrow) and the endothelial cell nuclei (arrowheads in **f**). For negative controls see Supplementary Fig. S2.

**Figure 5:**
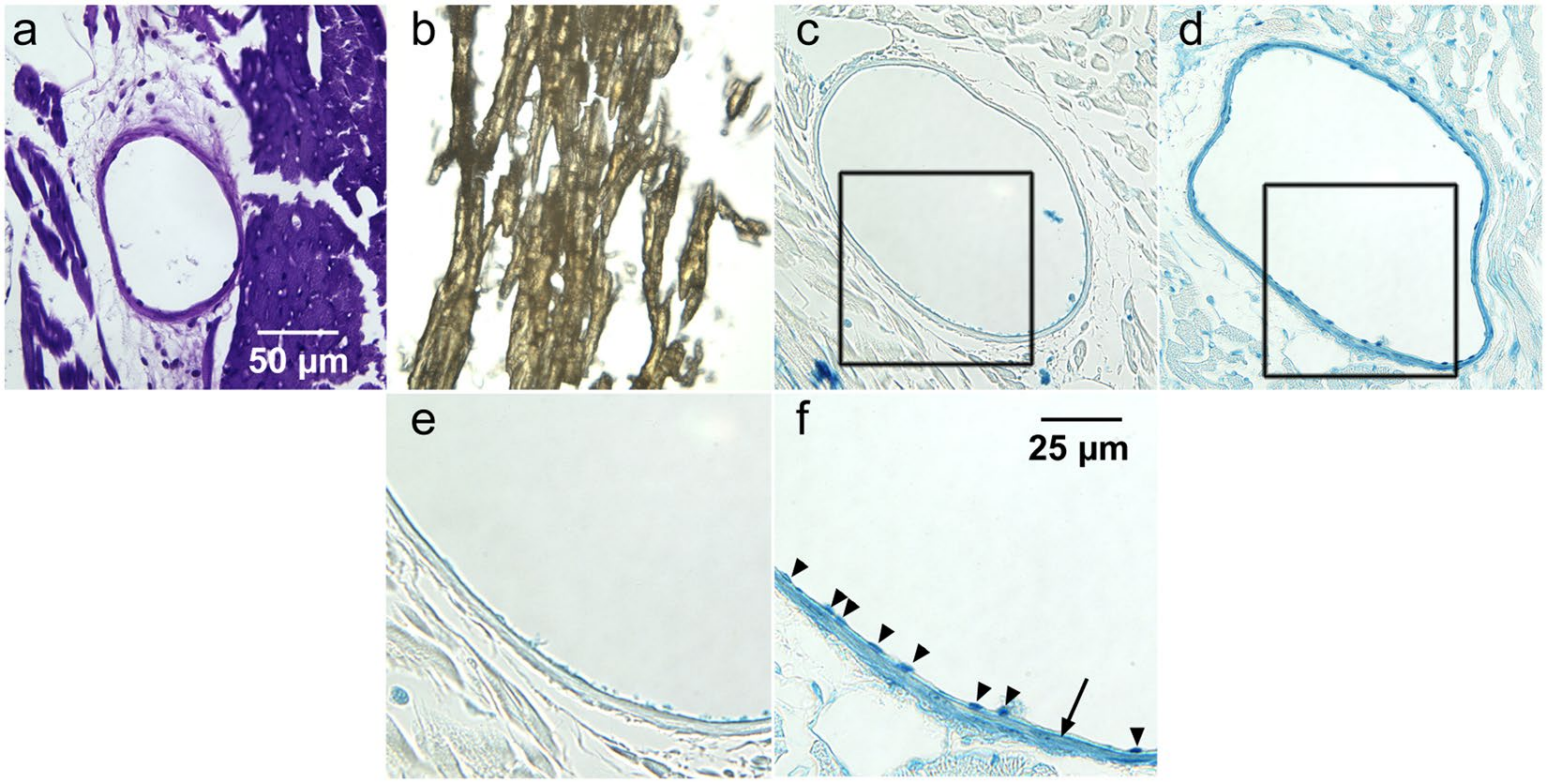
Sections of myocardium from mice perfused with PTA (**a**), I_2_KI (**b**), AlumHemFeI (**c**) and AlumHemFeI-T (**d**). The images in (**e**) and (**f**) are magnifications of the regions outlined in c, and d respectively. The purple colour in (**a**) indicates the presence of PTA within the myocardium and coronary wall. Intense iodine staining of the myocardium is seen in (**b**) (frozen section). Intense Prussian blue reaction product is seen in the coronary wall (**d** and **f**) when AlumHemFeI–T is used. The endothelial and medial layers (arrow in **f**) and endothelial cell nuclei (arrowheads in **f**) appear intensely stained. For negative controls see Supplementary Fig. S3.

To relate the micro-CT images to histology, sections of excised aortas and hearts from perfused mice were examined. Light microscopy images of representative histological sections of heavy element stain-perfused mice are shown in Figs 4 and 5 for the aorta and myocardium/coronary arteries, respectively. Negative-control histological sections are of aorta and myocardium are provided in Supplementary Figs S2 and S3, respectively; these figures contain histological images of all CT stains evaluated. In each case, the negative-control sections appear colourless, verifying that any pigmentation in the CT stain-perfused tissues was due to the presence of the CT stain.

In PTA-perfused sections, subsequent staining with toluidine blue marked the presence of PTA throughout the aortic wall, including the endothelium, the medial interlamellar units (between the elastic laminae) and adventitia (Fig. 4a). Within the myocardium (Fig. 5a) high intensity PTA-detection staining was observed in both the coronary vessel walls and the surrounding myocardial tissue. This observation is consistent with the micro-CT image (Fig. 2a) and indicates the delivery of the PTA stain into the myocardium, via the coronary vessels and capillaries.

In I_2_KI-perfused mice, iodine stains tissues brown. The brown colour was uniformly distributed across the wall of the aorta (Fig. 4b) and high-intensity colour is seen throughout the myocardium (Fig. 5b), while no coronary arteries were detectable. (Note, the poor quality of the micrographs is because, to avoid washing away of the tissue-associated iodine, frozen tissue sections maintained in OCT embedding media needed to be studied and imaged. Nonetheless, Figs 4b and 5b confirm that the CT enhancement seen in Fig. 2b is due to I_2_KI accumulation within the entire aortic wall and the myocardium.

Figures 4c,d and 5c,d suggest that, interestingly, AlumHemFeI and AlumHemFeI-T stain specific components of the vessel wall. The Prussian blue reaction product showed the distribution of iron (in the aluminum, iodine, and iron heavy element mixture) as different intensities of blue. For AlumHemFeI, iron was present within the interlamellar units of the aorta (Fig. 4c,e and Supplementary Fig. S2), with the highest concentration in the endothelium (Fig. 4e). Particularly noteworthy was the finding of intense blue also seen within some endothelial-cell nuclei (Fig. 4e and Supplementary Fig. S2). Within the heart (Fig. 5c,e and Supplementary Fig. S3), very little iron signal was observed in the myocardium and the wall of the coronary artery, but again with the highest concentration in the endothelial lining of the coronary.

The sections from AlumHemFeI-T-perfused mice indicate that a substantial amount of iron was present across the entire aortic wall (Fig. 4d and Supplementary Fig. S2), with particularly striking signal in the endothelial cell nuclei (Fig. 4f). Iron was also present in the interlamellar units, especially the innermost unit, as well as the adventitia (Fig. 4d,f). Figure 5d shows the myocardium and representative coronary artery, where the blue reaction product is present in the endothelial and medial layers of the coronary wall, again with remarkably intense staining of the endothelial cell nuclei (Fig. 5d,f).

These staining profiles for the AlumHemFeI- and AlumHemFeI-T-perfused mice, in both aorta and coronary artery, suggest a notable enhancement of stain delivery, including into the endothelial cell nucleus itself, when the detergent Triton™ X-100 is employed in the stain delivery protocol.

## Discussion

In this study, we have shown that a novel, highly x-ray attenuating stain, AlumHemFeI-T, delivered via vascular perfusion, allows for whole-body *ex vivo* visualization of the vessel wall over the entire vascular tree in mice using micro-CT. The perfusion/staining technique promises to provide valuable information to pre-clinical cardiovascular studies due to the ease of staining and wide availability of laboratory micro-CT scanners. Optical histology verified that AlumHemFeI-T has an affinity for endothelial cells and the contents within the interlamellar units in the aorta and the media of the coronary arteries. Vessels were also clearly visible in the CT images of the cerebral vasculature and of highly vascularized organs such as the liver and kidney. The AlumHemFeI stain is inert and has no known toxic properties, unlike the osmium tetroxide stain that has previously been used to study the coronary arteries in mice via whole-body perfusion^22^.

Although an original consideration for this study was the potential to stain elastin, perfusing the animals with traditional Verhoeff’s elastin stain resulted in intravascular precipitation, limiting adequate perfusion. Some of the Fe^3+^ in Verhoeff’s staining solution oxidizes heamatoxylin to hematein, and is reduced to Fe^2+^. The hematein and iron then form complexes that bind to all components of tissues. The iodine in the solution may, by an unknown mechanism, increase the entry of iron-hematein complex into elastin, which is a hydrophobic protein. After immersion in Verhoeff’s solution, tissue sections are partly destained in aqueous ferric chloride, which extracts iron-hematein from cytoplasm and collagen, leaving the black complex in cell nuclei, elastic fibers and laminae, and myelinated nerve fibers^32^. When Verhoeff’s solution was perfused through mice, precipitates, probably from interaction of proteins and inorganic salts with the ethanol solvent, blocked the microvasculature. This rendered traditional Verhoeff’s incompatible with whole body perfusion and necessitated modification of the stain. We successfully avoided vessel blockage by replacing the alcoholic heamatoxylin used in the traditional Verhoeff’s stain with aluminum (Harris) heamatoxylin (AlumHemFeI). Although the AlumHemFeI stain does not bind to elastin, it may be used to provide a surrogate stain of elastin by binding to components in the interlamellar units. The AlumHemFeI stain achieved more micro-CT contrast enhancement of all vessel walls than I_2_KI perfusion, especially those of the coronary arteries and aorta.

Further contrast enhancement was achieved when perfusion with AlumHemFeI CT stain was preceded by flushing with a saline solution of the non-ionic detergent Triton™ X-100, resulting in greater permeation of the stain into the vessel wall. We suspect that the saline-Triton™ X-100 solution allowed access to binding sites in the tissues that would not normally be accessible to the stain. The use of Triton™ X-100 also resulted in sharper delineation of the vessel wall in the micro-CT images, likely because of the increased permeability of the vessel wall to the final saline flush, thereby removing any excess stain that is present in surrounding tissue.

Based on the AlumHemFeI-T histology and the micro-CT results (Figs 2, 4, 5 and Table 1), we speculate that the non-ionic detergent is acting in two ways. First, the cell membranes of endothelial tissue are disrupted when exposed to the detergent, thereby allowing more stain to be delivered into the vessel wall. This assumption is supported by the more localized enhancement of contrast in the vessel wall compared to the surrounding tissue in the micro-CT images and the increased intensity of the blue colour observed in the Prussian blue-stained histology sections (compared to those stained with AlumHemFeI). Second, the increased permeability allowed for a more effective flush of the CT stain during the final saline perfusion stage; excess CT stain that wasn’t bound to tissue was removed resulting in more uniform enhancement of the vessel walls and sharper vessel wall delineation in the micro-CT images. The observed CT contrast is lower because of the partial-volume effect (where the CT voxels are larger than the thickness of the vessel wall).

Other groups have reported that using a graded ethanol biochemical conditioning technique enhanced the permeability of murine tissues^29^ and provided good soft tissue contrast without the use of a CT stain^23^. However, it has also been shown that when applied to both formalin-fixed and unfixed collagen-containing tissue, ethanol causes large amounts of shrinkage^36–38^. Our results show that using a biological detergent prior to perfusion of unfixed tissue permeabilizes the cell endothelial membrane and potentially opens channels in other tissue components to allow for greater stain penetration without the drying effects of ethanol.

AlumHemFeI and AlumHemFeI-T contain three highly attenuating elements – iron, iodine, and aluminum. The histological study shows that iron is present in select tissues and we have used this information to confirm the presence of the CT stain in those tissues. However, further analysis is needed to determine which of the three attenuating elements are dominantly contributing to the micro-CT contrast. Preliminary studies where mice were perfused with combinations of the three stain ingredients (FeCl_3_, I_2_KI, and Harris heamatoxylin) demonstrated CT enhancement only when iodine was present in the perfusate (results not shown), suggesting that most of the CT attenuation may be caused by iodine. In future studies, the actual amount of each elemental component in the various tissues will be determined using inductively coupled plasma mass spectrometry (ICP-MS), which is capable of detecting the presence of aluminum, iron and iodine in tissue.

Soft tissue staining with various CT stains can provide morphological information and has been explored extensively by soaking of tissues and organs. Our group pioneered the use of vascular perfusion to target the delivery of the CT stain to vascular tissue – by taking advantage of the inherent permeability of fresh, unfixed tissue to the small molecules in the CT stains – while simultaneously speeding up preparation from days to minutes^24^. In the earlier study, we demonstrated that PTA stains the vasculature, while I_2_KI is useful for the study of cardiac tissue. We now provide clearer understanding of the localization of each of these stains. PTA enhanced the contrast of the myocardium, aorta and the parenchyma of the liver in the micro-CT images (Fig. 1). This observation is consistent with previous findings that have demonstrated the affinity of PTA for collagen and the extracellular matrix^34,25^, and is further validated by the histological analysis (Figs 4 and 5). Although the presence of PTA in the vessel walls and heart is evident, the CT enhancement is lower than that seen with other CT stains evaluated (I_2_KI, AlumHemFeI, and AlumHemFeI-T). I_2_KI provides greater CT contrast enhancement of the myocardium and ascending aorta, allows visualization of the myocardial fibres and provides a negative lumen stain of the coronary arteries in micro-CT images.

In this study, we also introduce the use of a controlled CT-stain perfusion system, which enables physiological pressurization and precise timing of all the steps of stain delivery. While similar results can likely be achieved using hand-perfusion or a gravity-fed IV set up, the use of timed perfusion and known pressure resulted in higher reproducibility of the results. Furthermore, in this study, animals were perfused using a retrograde abdominal aortic procedure with cannulation occurring just below the kidneys. This route was selected to enable the visualization of the coronary as well as the thoracic vasculature, although it does not allow for the lower abdominal region to be perfused. Alternative stain delivery routes, such as left ventricular puncture or antegrade abdominal aortic perfusion would allow for other vascular systems to be perfused and studied. Many disease models that involve the changes or alterations in the blood vessel wall (*e.g*. aneurysm models, atherosclerosis models, cancer, angiogenesis, and vascular grafts) would benefit from this technique^2,5,7^.

Larger animals are also commonly used in studies of vascular disease and we expect that the perfusion with AlumHemFeI-T, as described in this work, will be equally applicable to such studies. The perfusion technique uses the vasculature as a conduit to deliver the stain to the vessel walls and tissues, which can have varying properties across different species. Hence, some optimization to the perfusion times may be required in different animal species, body weights, or tissue type. Nevertheless, preliminary perfusion of a guinea pig, an adult rat and an explanted porcine heart demonstrated similar results as those observed in mice. (Supplementary Fig. S4 shows an image of the perfused rat). While not demonstrated in this study, the presented virtual histology technique also allows for high-resolution non-destructive imaging of tissues excised from the perfused animals (*i.e*. sectioned post fixation). However, further investigation is required to determine if stain diffusion over long fixation and scan periods will affect stain localization.

Our rapid perfusion and staining technique allows for the heavy element stains to be quickly and evenly distributed throughout the tissues addressing limitations that other groups have faced and allowing for larger sample sizes to be stained and studied. Moreover, tissues stained with AlumHemFeI-T can then be harvested from the intact mouse and further processed histologically, an important advantage over other CT staining strategies that allows for multi-modality assessment of the vasculature.

## Conclusions

We have developed and demonstrated a novel non-toxic stain – AlumHemFeI-T – that provides excellent visualization of the vasculature of mice for virtual histology, with unique sensitivity to endothelial nuclei as well as the media of large and medium size vessels. Delivered using a novel perfusion apparatus, the AlumHemFeI-T stain represents a promising new tool for scientists studying both normal and diseased vasculature in rodent models.

## Supplementary information

Supplementary figures

Axial_slices_video

Sagittal_slices_video

Coronal_slices_video

## Data Availability

Data sets are available upon request from the corresponding author.
