# Supplementary material for: 3D vessel-wall virtual histology of whole-body perfused mice using a novel heavy element stain
Source: Sci Rep. 2019 Jan 24;9:698. doi: 10.1038/s41598-018-36905-z (PMC6345940; doi:10.1038/s41598-018-36905-z)
Supplement: Supplementary file 1 — Supplementary figures [file 41598_2018_36905_MOESM1_ESM.pdf]

Supplementary Figures: 3D vessel-wall virtual histology of whole-body perfused mice using a novel heavy element stain

P. Joy Dunmore-Buyze, Charmainne Cruje, Zengxuan Nong, Jason J. Lee, John A. Kiernan, J. Geoffrey Pickering, and Maria Drangova

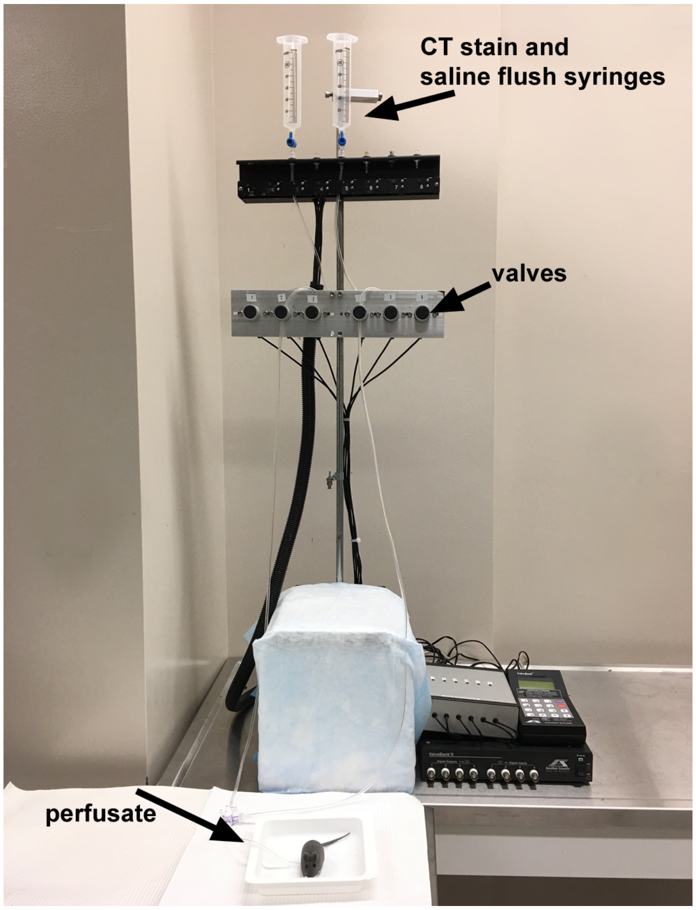

Figure S1. The CT stain perfusion system consists of a modified ValveBank®8 Pinch Valve (Automate Scientific, Berkeley, CA) gravity-fed perfusion system, which seamlessly switches between solutions. The pinch valve apparatus was redesigned to allow for the use of larger diameter tubing (4.76 mm), which provided the flow rates and pressure required to perfuse whole animals. Up to 6 different solutions can to be perfused, as required, without moving the animal once the catheter is in place.

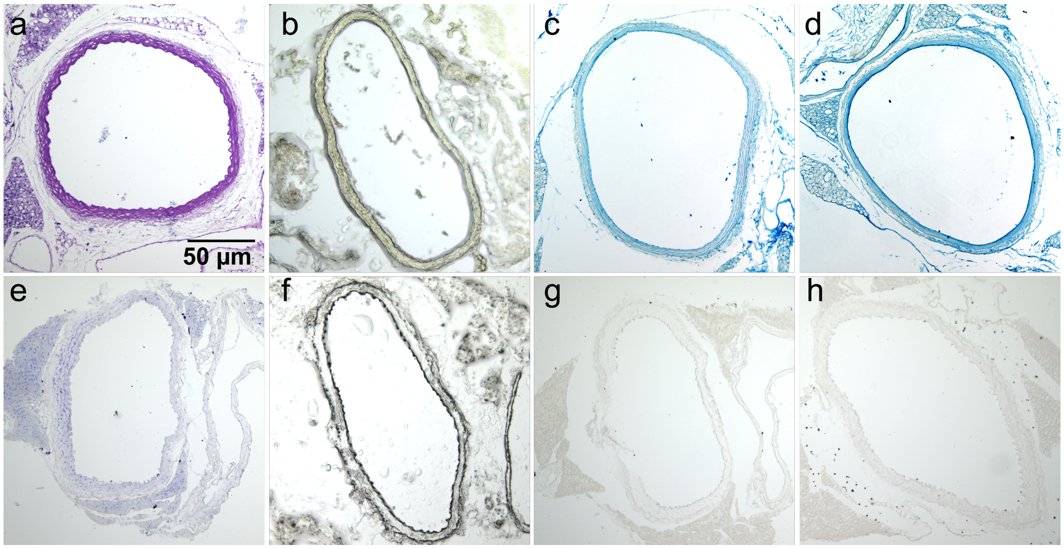

Figure S2. Sections of aortas from mice perfused with PTA (a), I_2KI (b), AlumHemFeI (c) and AlumHemFeI-T (d). Corresponding negative controls, *i.e.* sections from mice perfused with saline then treated with the same counter stains (where needed) are show in in e through h.

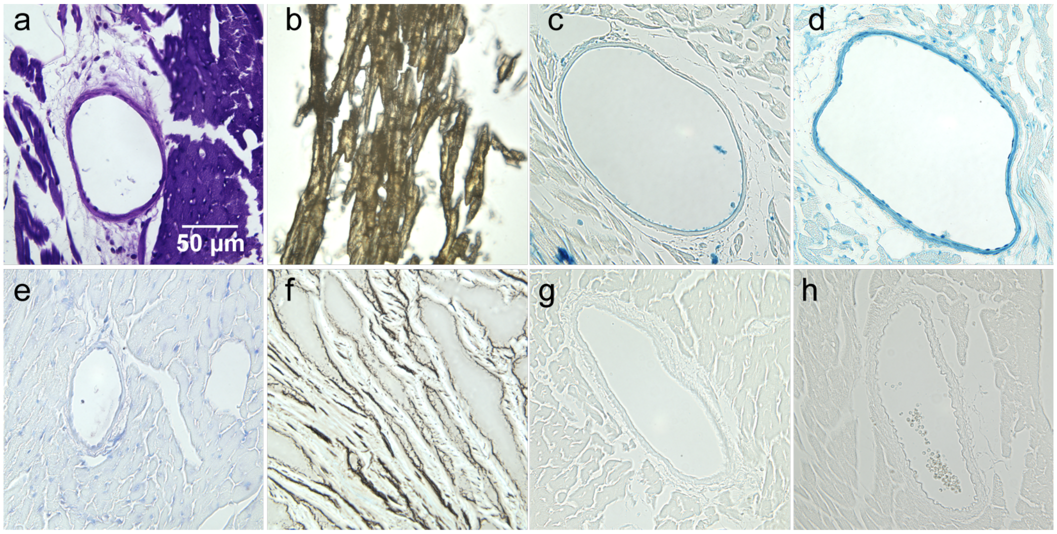

Figure S3 Sections of myocardium and coronary vessels from mice perfused with PTA (a), I_2KI (b), AlumHemFeI (c) and AlumHemFeI-T (d). Corresponding negative controls, *i.e.* sections from mice perfused with saline then treated with the same counter stains (where needed) are show in in e through h.

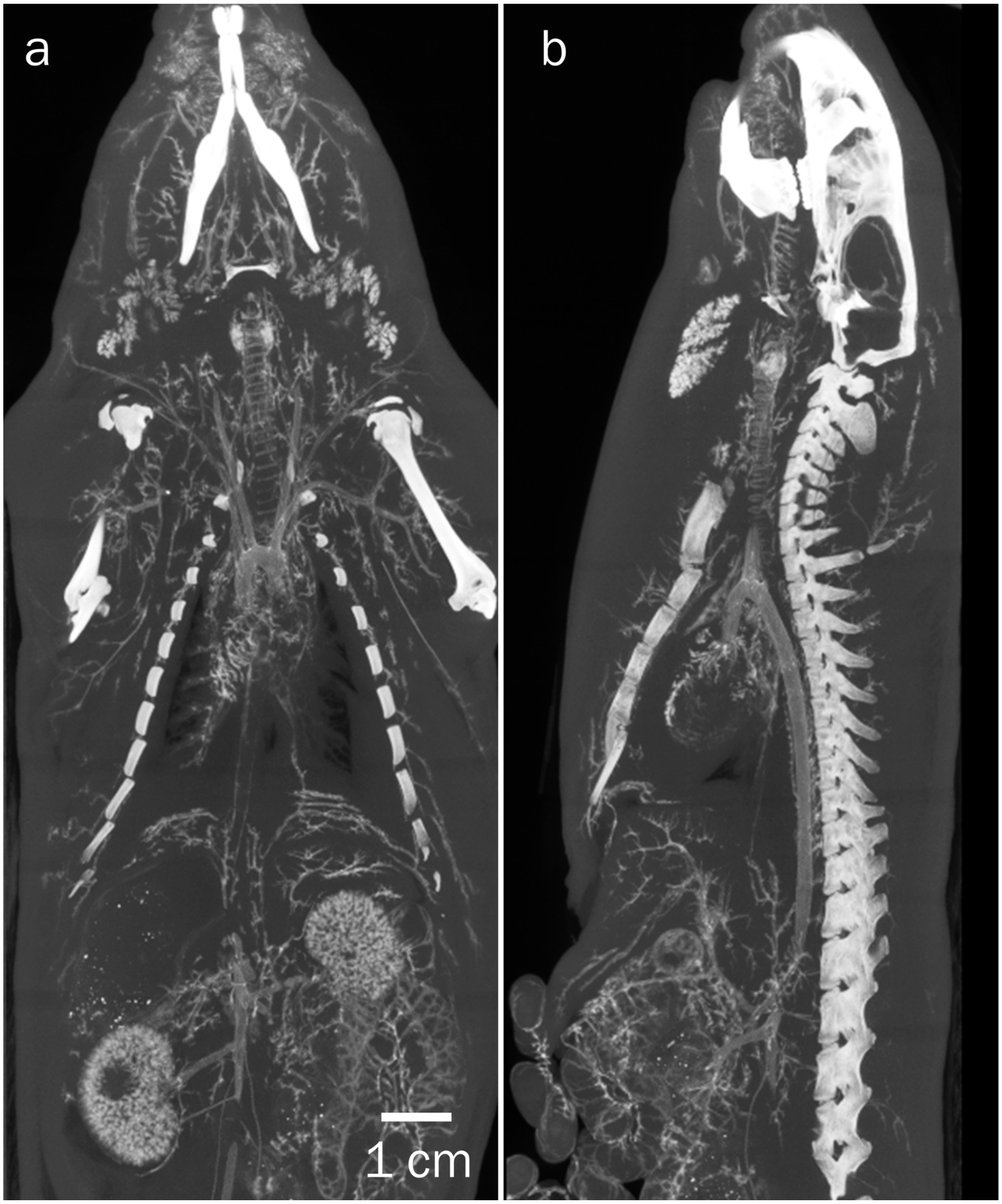

Figure S4 Coronal (a) and sagittal (b) maximum intensity projections (5-mm thick) from a single adult rat that was perfused with the AlumHemFeI-T stain. The image was acquired with a 5-minute scan protocol (90 kVp; 50 µm voxels).
